# Action-Based Encoding Improves Instruction Following in Children and Adolescents

**DOI:** 10.3390/bs16061008

**Published:** 2026-06-16

**Authors:** Zhaotong Yao, Xiaomin Su, Yuxi Zhao, Richard J. Allen, Amanda H. Waterman, Tianxiao Yang

**Affiliations:** 1 Neuropsychology and Applied Cognitive Neuroscience Laboratory, State Key Laboratory of Cognitive Science and Mental Health, Institute of Psychology, Chinese Academy of Sciences, Beijing 100101, China; yaozt@psych.ac.cn (Z.Y.); suxm6@mail2.sysu.edu.cn (X.S.); zhaoyuxi24@mails.ucas.ac.cn (Y.Z.); 2 Department of Psychology, University of Chinese Academy of Sciences, Beijing 100049, China; 3 School of Psychology, University of Leeds, Leeds LS2 9JT, UK; r.allen@leeds.ac.uk (R.J.A.); a.h.waterman@leeds.ac.uk (A.H.W.)

**Keywords:** following instructions, rehearsal, observation, imagery, enactment

## Abstract

Encoding and recalling spoken instructions play a key role in successful learning in the classroom. Previous research in adults suggests that, relative to simple verbal rehearsal, three forms of action-based encoding (i.e., motor imagery, action observation, or self-enactment) facilitate instruction recall to a similar extent. This study aimed to examine whether motor imagery, action observation, and self-enactment could improve memory for instructions in children and adolescents, and to compare the effectiveness of these strategies. In Experiment 1, children aged 8 and 9 years listened to instructional sequences that varied in length (2, 3 and 4 actions) while using one of the encoding techniques (i.e., motor imagery, action observation, self-enactment, or verbal rehearsal), followed by oral repetition or enacted recall. In Experiment 2, adolescents between age 12 and 14 were tested using a similar design except that the instructions were all four-action sequences. In Experiment 1, for both verbal and enacted recall, children’s memory performance in each of the three action-based encoding conditions was superior to the rehearsal condition, although the benefit from motor imagery was relatively smaller. In Experiment 2, adolescents displayed similar patterns as children, except that motor imagery yielded a stronger and more reliable advantage in this age group. The current findings suggest that, for both children and adolescents, encoding spoken instructions by imagining, observing, or performing the actions yields comparable mnemonic advantages, and thus provides practical ways for supporting and enhancing working memory in classroom environments.

## 1. Introduction

Understanding effective strategies to support working memory and avoid cognitive overload in the classroom is critical for research on working memory and its application to educational contexts (e.g., Atkinson et al., 2021; Gathercole & Alloway, 2008; Sweller et al., 2019). One important way of learning is by following instructions, and teachers often use spoken instructions to guide students in the classroom. Research has shown that difficulties with instruction following can impair students’ learning (Gathercole et al., 2008) and acquisition of academic skills such as mathematics (Gilmore et al., 2024).

When children memorize spoken instructions, repeating the instructions (i.e., verbal rehearsal) is a common way of maintaining the information in working memory. Alternatively, recent studies indicate that involving action-based processing at encoding (e.g., self-enactment, action observation, and motor imagery) and retrieval (e.g., recall by enactment) can be effective in improving instruction following in children (see Allen et al., 2023, a review; Gathercole et al., 2008; Jaroslawska et al., 2016; Makri & Fiske, 2023; Waterman et al., 2017; Xie et al., 2022; T. Yang et al., 2017, 2021). This study aims to compare the efficacy of these action-based encoding strategies in order to deepen our understanding of the facilitating and hampering factors for following spoken instructions in children and adolescents.

### 1.1. Verbal Rehearsal

For children (Tam et al., 2010) and adults (e.g., Morrison et al., 2016), verbal rehearsal is a commonly implemented strategy for retaining verbal information in working memory. Under the framework of Baddeley’s multicomponent working memory model (Baddeley, 1992; Baddeley et al., 2021), the phonological loop is responsible for maintaining verbal information, while the rehearsal process serves to maintain/refresh auditory information from decay and recode non-auditory information into phonological form. In terms of spoken instructions, previous research has already recognized the critical role of verbal working memory (possibly via rehearsal) in preserving the instructional content in children and adults (Engle et al., 1991; Gathercole et al., 2008; Jaroslawska et al., 2016, 2018; Li et al., 2022; T. Yang et al., 2016).

Regarding the development of this strategy, children younger than 7 years generally do not spontaneously use subvocal rehearsal, but they can learn to use this strategy after training (Johnston et al., 1987). From age 7, children begin to use verbal rehearsal, and this ability and this skill continue to improve throughout middle childhood and into adolescence (Bruns et al., 2019; Gathercole, 1998; Tam et al., 2010). Overall, these findings suggest that typically developing school-age children and adolescents can use rehearsal for maintaining verbal information.

### 1.2. Self-Enactment

Self-enactment is another useful strategy, which has been extensively investigated in research on the long-term retention of actions (e.g., Engelkamp & Zimmer, 1989; Kormi-Nouri et al., 1994; Mohr et al., 1989; Norris & West, 1993; Zimmer et al., 2000). When participants were required to remember a long list of action–object phrases, their memory performance was usually better when they acted out the actions than simply read them (Engelkamp & Zimmer, 1989). The self-enactment effect is considered to arise from encoding additional motoric information (Engelkamp & Zimmer, 1985), multimodal information within a rich context (Bäckman, 1985; Bäckman & Nilsson, 1984), greater self-involvement and integration of actions, objects, and self-environment (Kormi-Nouri, 1995), enhanced item-specific processing due to combined conceptual knowledge with physical action (Engelkamp, 2001), as well as strategic- and goal-related benefits (cf. Roberts et al., 2022). In terms of the neural mechanism, the self-enactment effect reflects enhanced neural activation in the parietal lobes (e.g., supramarginal gyrus) and frontal lobes (Ma et al., 2021; Russ et al., 2003). The facilitative effects of self-enactment on long-term memory appear around age 4–6 years (J. Yang & Wang, 2021), and increase across childhood and adolescence (Badinlou et al., 2017, 2018; Ratner & Hill, 1991; Söderlund et al., 2023).

The self-enactment effect is also present in the working memory domain. Physical performance of instructed action steps during the presentation of spoken instructions improved subsequent recall among adults (Allen & Waterman, 2015; Charlesworth et al., 2014; Lui et al., 2018) and, as long as the task was not too complex, in children (Jaroslawska et al., 2016; Waterman et al., 2017; Wojcik et al., 2011). As well as supporting encoding, an enactment benefit has also been consistently observed at retrieval (see Allen et al., 2023); memory for instructions is superior when they are executed physically than orally repeated at retrieval, both in children (Gathercole et al., 2008; Jaroslawska et al., 2016; T. Yang et al., 2017) and adults (e.g., Allen & Waterman, 2015; Koriat et al., 1990; T. Yang et al., 2014).

Recent findings involving adult participants indicate that the enacted-recall advantage may stem from motor planning for enacted recall (Jaroslawska et al., 2018; Li et al., 2022), as it facilitates binding of to-be-performed actions and objects in the environment (Makri & Jarrold, 2021; T. Yang et al., 2016). Interestingly, the self-enactment benefit at encoding is often reduced or removed when recall is also through enactment (Allen & Waterman, 2015; Jaroslawska et al., 2016; Waterman et al., 2017). The finding can be explained by the overlap of motoric representations, that is, when participants are executing actions when encoding spoken instructions, or preparing for later enacted recall, they tend to develop explicit action plans (Koriat et al., 1990). Consequently, when the instructions have already been enacted when encoding spoken instructions, action planning to prepare for enacted recall is no longer needed nor useful, because the motoric memory trace generated via physical enactment has already been utilized.

### 1.3. Action Observation

A common process by which people learn is through observing others’ demonstrations (Burke et al., 2010). For example, observational learning for procedural and motor skills is frequently used in physical education (Han et al., 2022), driving lessons (Deppermann, 2015) and surgical training (Cordovani & Cordovani, 2016; Mentis et al., 2014; Rao et al., 2023). In long-term memory, recall of actions is better when those actions were demonstrated by others compared with when they were verbally presented at encoding (Cohen, 1983; Engelkamp & Dehn, 2000). This advantage has consistently been observed in developing populations (Badinlou et al., 2017, 8–14 years; J. Yang & Wang, 2021, 4–6 years), and relates to the benefits from multimodal encoding and improved episodic integration.

Demonstration often involves providing a series of verbal instructions that outline the successive steps required to complete a specific task, coupled with a manual demonstration of the actions for each step. In instruction following research within a working memory paradigm, various demonstration formats have been investigated. In some studies, silent video demonstration was used, which improved recall substantially over purely auditory presentation of verbal instructions in both children (T. Yang et al., 2017) and adults (T. Yang et al., 2015, 2019, 2022). In other studies, demonstration of actions occurred simultaneously with the spoken name of the action (Lui et al., 2018; T. Yang et al., 2015, 2022) or immediately after it (Waterman et al., 2017; T.-X. Yang et al., 2024). In all cases, the inclusion of action demonstration led to substantial memory benefits. This advantage arises from the inclusion of additional visual and spatial information, which helps form richer and multimodal memory representation more robustly than verbal-based representation (Allen et al., 2020). Notably, while most studies showed demonstration enhanced both verbal and enacted recall (Lui et al., 2018; Waterman et al., 2017; T. Yang et al., 2015; T.-X. Yang et al., 2024), occasional exceptions exist where it failed to improve enacted recall (T. Yang et al., 2017, 2022).

In long-term memory, the demonstration benefit (often termed the experimenter-performed task benefit, EPT) is often compared with self-enactment (termed the self-performed task benefit, SPT). According to multimodal encoding theory (Engelkamp, 2001), while both action-encoding benefits rely on multimodal information, SPT predominately facilitates item-specific processing while EPT tends to facilitate item-relational processing. A review by Steffens et al. (2015) indicated that SPT outperforms EPT in recognition tests and free recall of long lists or mixed lists involving both encoding types, reflecting a relative advantage of item-specific processing over item-relational processing. For instance, long action lists may diminish the item-relational processing advantage associated with EPT. In contrast, comparable performance between SPT and EPT conditions is observed in free recall of short lists, pure lists, and action sequences. This may be due to the trade-off effect between item-specific and item-relational processing. Meanwhile, it reflects shared representations underlying action perception, planning, and execution, which aligns with common coding theory (Prinz, 1997). Nevertheless, a recent meta-analysis indicates an overall memory advantage of SPT over EPT (*g* = 0.51), suggesting an additional benefit of motor coding during self-enactment (Roberts et al., 2022).

Compared to the complex and lengthy action sequences examined in the long-term memory research, those in the working memory domain are typically shorter and allow maintenance and processing within a limited time frame. Several studies using following instruction tasks have found demonstration to be a superior and more stable method for improving memory of instructions (Allen et al., 2020; Coats et al., 2021; Waterman et al., 2017). However, other studies suggest self-enactment results in better memory performance than demonstration (Lui et al., 2018; Wojcik et al., 2011), while one study reports similar performance levels (T.-X. Yang et al., 2024). Such inconsistent findings imply that, just as in the long-term memory domain, the advantages of self-enactment and demonstration each exert unique strengths in working memory, and their relative advantages may therefore vary across different contexts and populations.

### 1.4. Motor Imagery

Motor imagery is a dynamic process whereby individuals mentally simulate action sequences in their working memory without producing any overt movements (Caeyenberghs et al., 2009b; Decety & Grèzes, 1999). Motor imagery not only creates an internal and forward model to predict the progression of upcoming action execution (Wolpert, 1997), it also recruits frontoparietal and subcortical brain networks to simulate the complex process of concrete action execution (Hétu et al., 2013). Motor imagery ability develops during childhood from age 5 to 12 years, with gradual refinement into adolescence (Caeyenberghs et al., 2009b; Smits-Engelsman & Wilson, 2013; Souto et al., 2020; Spruijt et al., 2015).

In the following instruction domain, more recent research has attempted to examine the potential benefit of imagination of actions during encoding. T. Yang et al. (2021) explicitly asked child participants to imagine performing the instructions during presentation and found this strategy improved recall performance compared to verbal encoding. This provides evidence for the benefits of forming an action-based representation via motor imagery in a developmental population. Moreover, motor imagery yielded similar benefits in the enacted and verbal recall conditions, suggesting additional benefits above action planning for enacted recall. This pattern of motor imagery superiority over verbal rehearsal across verbal and enacted recall has also been replicated in adult participants (T.-X. Yang et al., 2024).

### 1.5. Research Gap and the Present Study

In the domain of long-term memory, action advantages have been investigated over many years (Zimmer et al., 2001; Roberts et al., 2022), while exploration in the working memory context is relatively recent. Research to date in the working memory literature has consistently shown an enacted-recall advantage (see a review by Allen et al., 2023). In terms of action-encoding advantages (e.g., action observation, self-enactment, motor imagery), their efficacy is evident when compared to various control conditions, including passive listening to instructions (e.g., Allen et al., 2020, Experiment 1; Allen & Waterman, 2015; Coats et al., 2021; Jaroslawska et al., 2016; Waterman et al., 2017; T. Yang et al., 2015, Experiment 2), verbal rehearsal (T. Yang et al., 2021; T.-X. Yang et al., 2024), and an attentionally demanding condition (Xie et al., 2022).

In comparison, research examining the benefits of different action-encoding techniques on following instructions remains limited (Coats et al., 2021; Lui et al., 2018; Wojcik et al., 2011; Xie et al., 2022; T.-X. Yang et al., 2024); and as aforementioned, findings regarding the relative advantages of these action-encoding methods are inconsistent. Until now, only one study has directly compared all three types of action encoding (i.e., motor imagery, action observation, self-enactment) in adults (T.-X. Yang et al., 2024), and importantly, none have done so with child populations. Further, there have been no studies on adolescence. Investigating how different forms of encoding techniques affect following instruction performance across childhood and adolescence will enhance our understanding of optimal strategies to improve working memory for instructions in these age groups within educational settings.

Accordingly, the main purpose of this study is to directly compare the benefits of motor imagery, action observation, and self-enactment, relative to a verbal rehearsal strategy in typically developing children (Experiment 1) and adolescents (Experiment 2). Across the two experiments, we used a typical following instruction task that has been adapted from previous versions (Gathercole et al., 2008; T. Yang et al., 2021; T.-X. Yang et al., 2024), which requires the short-term retention of spoken instruction sequences and relies on working memory (Jaroslawska et al., 2018; T. Yang et al., 2014, 2016). In this study, across different conditions, participants memorized the spoken instruction sequences using each encoding method in turn, including the verbal rehearsal, motor imagery, action observation, and self-enactment strategy. During the retrieval stage, participants recalled the entire instruction sequence via repetition or enactment. The hypotheses are presented in each experiment.

## 2. Experiment 1

In this experiment, we compared children’s performance in following spoken instructions under four encoding conditions (i.e., verbal rehearsal, motor imagery, action observation, and self-enactment). Firstly, as repeatedly demonstrated in the literature (see Allen et al., 2023, for a review), an enacted-recall advantage was anticipated in the present study. Secondly, given previous evidence of action-encoding advantages relative to verbal rehearsal (T. Yang et al., 2021; T.-X. Yang et al., 2024), we expected performance in the motor imagery, action observation, and self-enactment conditions would be better than that in the verbal rehearsal condition. Finally, regarding comparisons among the three forms of action-encoding techniques and interactions between encoding- and retrieval-based action advantages, research remains limited with inconsistent findings; accordingly, no specific hypotheses were formulated for these aspects.

### 2.1. Methods

#### 2.1.1. Participants

In order to estimate the sample size required for detecting action-based advantages, G*Power 3.1 software was used (Faul et al., 2009). The estimate was based on the T.-X. Yang et al. (2024) study, in which the effects of action advantages at the encoding stage were averaged, resulting in Cohen’s *d* = 0.94. Detecting an effect of this magnitude in an *F* test with alpha = 0.05 and 80% power suggests the requirement for 24 participants. Using the same method, the estimated sample size for the enacted-recall advantage was 32. Therefore, 40 children from a randomly selected Grade 3 class in a primary school were invited to the study. One child had difficulty completing the task and was removed. The final valid sample included 39 children (21 girls, 18 boys), and their mean age was 8.46 years (*SD* = 0.51, from 8 to 9 years old). The present study was approved by the institute ethics committee of the corresponding author (H19052). All the parents of participants provided informed consent.

#### 2.1.2. Design

It was a 4 × 2 mixed design, and the within-participant variable was the encoding technique (i.e., verbal rehearsal, motor imagery, action observation and self-enactment), and the between-participant variable was the recall modality (i.e., verbal vs. enacted recall). The proportion of correct action–object pairs was the dependent variable. The order of encoding conditions was counterbalanced across participants.

#### 2.1.3. The Instruction Span Task and Procedure

**Figure 1:**
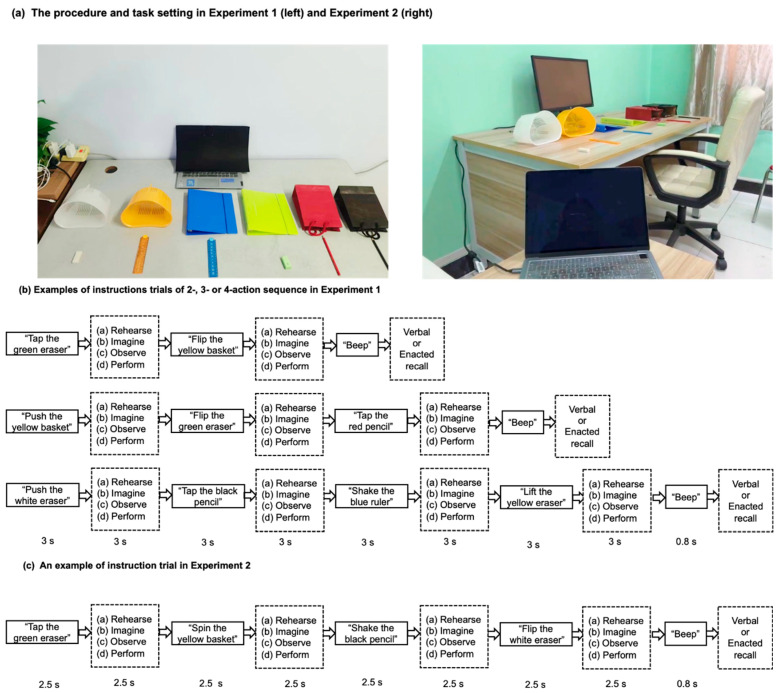
Display of objects and instruction trials in Experiments 1 and 2. In the two experiments, the oral instructions were presented in Chinese; the English words here are solely for procedure comprehension. In each trial, only one of the four encoding techniques (i.e., rehearse, imagine, observe, perform) was used.

The task was taken from T. Yang et al.’s (2021) study with a few adaptations. In short, the spoken instructions consisted of action–object sequences, including 6 types of actions (e.g., flip, spin, pull, shake, tap, lift), and 12 objects varied in colors (e.g., erasers, rulers, pencils, basket, folders and bags, see Figure 1).

The task comprised four instruction lists. In each list, there were three blocks and each consisted of four trials. In each block, the numbers of action–object pairs were the same. For instance, there were two action–object pairs in the first block (e.g., “tap the white basket, push the blue ruler”), and three action–object pairs in the second block (e.g., “flip the yellow ruler, shake the black pencil, lift the blue folder”), and so on. Objects and actions did not repeat within a trial. Each action–object pair was read by a native Chinese woman using a moderate speed and recorded as an audio file. In each spoken instruction sequence, each audio file was followed by a 3 s silence interval for participants to implement the assigned encoding strategy, and these audio files with silent intervals were edited to form a coherent sequence. These spoken instruction sequences were used for the verbal rehearsal, the motor imagery, and the self-enactment conditions. For each action–object pair, a person performing the action was also videotaped and used as the demonstration materials for the action observation condition. To make a coherent video file for each instructional sequence, an audio file was accompanied by a black screen (3 s) and followed by a short video of that action (3 s), and edited as a coherent video file.

Each child was tested in a quiet room of the school by an experimenter. In each condition, the child was seated at a desk with two arrays of objects in front and a computer screen positioned behind the object display. This arrangement was the same for every participant throughout the session. The experimenter first informed participants that they were going to be playing a game relating to memory. Next, the experimenter told the children how to name the 12 objects and 6 actions, and also demonstrated the correct ways to perform the actions. The children were then required to practice until they achieved complete accuracy.

Afterwards, the experimenter told the children that they were going to hear and remember instructional sequences, and then repeat them aloud or act them out. In addition, they would be taught a specific mnemonic strategy in each condition, and they were required to use that strategy to memorize the instructions.

Each trial began with the experimenter saying ‘prepare’. After a 500 ms silent interval, the first action–object pair was presented, followed by a 3 s silent period during which they could implement the just-learnt encoding strategy. The second action–object pair was then presented, followed by another silent time window for the implementation of the strategy, and so on. During the silent interval of encoding, participants applied different encoding strategies across conditions: they quietly repeated the instructions in the verbal rehearsal condition, imagined themselves performing the actions on the objects in the motor imagery condition, watched the video clip of the action demonstration in the action observation condition, or performed the action in the self-enactment condition. A beep signaled the end of an instruction sequence, at which point children began to recall by reproducing the sequences in their original serial order. In the verbal recall conditions, participants serially repeated the instructional sequence aloud, whereas in the enacted recall conditions, they sequentially executed the designated actions on the objects. In both recall conditions, participants were required to say “blank” for any action–object pair they could not remember.

An action–object pair was considered as correct (score = 1) when both features (i.e., action and object) were recalled accurately and placed in the correct serial positions. The total number of correct action–object pairs was then summed up and divided by 36 (i.e., the total action–object pairs in a list), which led to the *proportion correct of action*–*object pairs* of the list/condition (range: 0 to 1).

#### 2.1.4. Data Analysis Plan

All analysis was carried out in JASP (https://jasp-stats.org/, accessed on 5 February 2024). We applied a repeated-measures ANOVA on the overall 2 × 4 design. In addition to frequentist analysis, a Bayesian ANOVA was also implemented for the 2 × 4 models, with Bayes factors (BFs) reported as indices of how strongly the data favored the null versus the alternative hypotheses. Values of *BF* < 1 were interpreted as support for the null hypothesis, whereas *BF* > 1 indicated support for the alternative hypothesis. Bayes factors provide a graded measure of evidential strength, with *BF* 1–3 as anecdotal evidence, *BF* 3–10 as moderate evidence, and *BF* > 10 as strong evidence (Jeffreys, 1961; Lee & Wagenmakers, 2013).

### 2.2. Results

#### 2.2.1. Preliminary Analysis

There were no significant gender effects on the action–object pairs scores in all conditions, with all *p* values > 0.05. The order of encoding conditions did not significantly affect action–object pair performance (*p* = 0.129). Reliability of the action–object pairs across all trials in the instruction span task was acceptable, with Cronbach’s alpha = 0.763. 

#### 2.2.2. Action–Object Pairs

**Figure 2:**
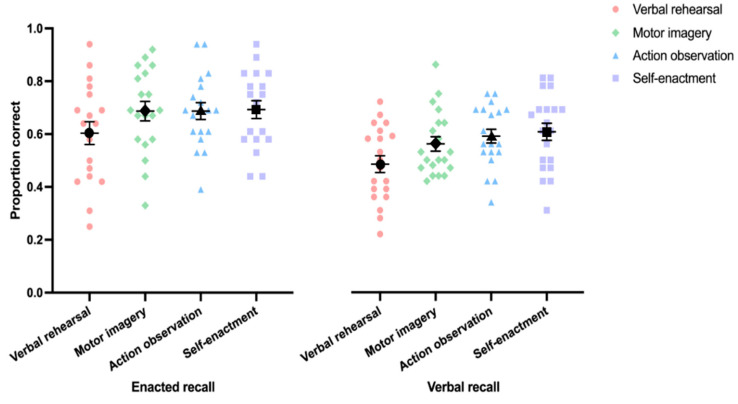
Proportion correct of action–object pairs as a function of encoding technique and recall modality in children.

Descriptive results of the action–object pairs are presented in Table 1 and Figure 2. A 4 (encoding technique: verbal rehearsal, motor imagery, action observation, self-enactment) × 2 (recall modality: verbal vs. enacted) mixed ANOVA was conducted. There was a significant main effect of encoding technique, *F*(3, 111) = 7.33, *p* < 0.001, *η_p_*^2^ = 0.17, *BF*_10_ = 138.74. Post hoc analysis with Bonferroni corrections indicated a similar level of performance among the motor imagery, action observation, and self-enactment conditions, with all *p* values > 0.05, while *BF*_10_ values were from 0.23 to 1.10. Superior performance was observed in the action observation and the self-enactment conditions relative to the verbal rehearsal condition (*p* = 0.009, *BF*_10_ = 45.19; *p* < 0.001*, BF*_10_ = 107.25, respectively). There was also a non-significant, weakly supported trend for the superior performance of the motor imagery condition relative to the verbal rehearsal condition, *p* = 0.075, *BF*_10_ = 1.82.

There was a significant main effect of recall modality, showing as superior enacted recall than verbal recall, *F*(1, 37) = 10.34, *p* = 0.003, *η_p_*^2^ = 0.22, *BF*_10_ = 10.65. The interaction between recall modality and encoding technique was not significant with a Bayes factor that supported the null hypothesis, *p* = 0.904, *BF*_10_ = 0.29. The effect sizes of the action advantages at encoding and retrieval are presented in Table 2 and Table 3.

### 2.3. Discussion

To our knowledge, this is the first study to directly compare memory for spoken instructions under the four encoding conditions of rehearsal strategy, motor imagery, action observation, and self-enactment in school-age children. Overall, we found benefits of action at both encoding and retrieval, and no interaction between these encoding- and retrieval-based effects. The three action-related conditions showed similar levels of performance (i.e., motor imagery, action observation, and self-enactment), with the latter two conditions being superior to verbal rehearsal. These findings are broadly in line with the findings in young adults (T.-X. Yang et al., 2024), and indicate similarity among the three forms of action-based encoding techniques.

Notably, however, compared to the clear benefit of action observation and self-enactment over verbal rehearsal, the evidence for motor imagery advantage seems weak. This may be because motor imagery requires more executive control than action execution (Glover & Baran, 2017), and children’s executive control and motor imagery skills are still developing (Fernández García et al., 2021; Spruijt et al., 2015). Therefore, encoding via action imagination may impose a cognitive cost during the encoding and retention of instructions—a cost that serves to reduce any benefits children can derive.

Aligned with this reasoning, previous findings indicate a developmental change in the advantage of motor imagery compared to verbal rehearsal in the instruction-following task. Specifically, the motor imagery benefit observed in school-age children (*d* = 0.35 in T. Yang et al., 2021, and 0.56 in the present experiment) was somewhat smaller than that in young adults (*d* = 0.67–0.91 in T.-X. Yang et al., 2024), which implies age-related enhancement in motor imagery skills and working memory capacity (Fernández García et al., 2021; Spruijt et al., 2015). Based on this speculation, adolescents should exhibit a greater motor imagery benefit than children, but a smaller one compared to adults. However, no study has investigated this issue in adolescents. Further investigation into the effects of motor imagery, along with action observation and self-enactment on instruction-following performance in adolescents would be valuable, which is the focus of Experiment 2.

In terms of self-enactment and action observation, the present findings indicate that the two mnemonic techniques yield similar levels of instruction-following performance in children. In the instruction following literature involving developing populations, only one small-scale study has directly compared the two techniques in a neurotypical sample, and these 7–16-year-olds showed greater benefits from self-enactment than from action observation (Wojcik et al., 2011). Waterman et al. (2017) investigated the two techniques in separate experiments involving children aged 6–10 years old. They found a large benefit of action observation, while the benefit of self-enactment was inconsistent and depended on sequence complexity. In a previous study on long-term action memory (Badinlou et al., 2017), 8-year-old children performed better in the action observation condition than in the self-enactment condition. The authors explained this may be because self-enactment consumes more limited cognitive resources than action observation when young children memorize lengthy action lists. Specifically, in their study, children learned three lists of action events, with each list containing 16 simple action phrases and lasting 166 s. In comparison, children completed immediate serial recall of short instruction sequences with fewer actions and objects in the following instruction task. Cognitive resource consumption induced by self-enactment during encoding was therefore limited and less disruptive, resulting in comparable performance between the two conditions.

In adults, the findings have also been inconsistent, with different studies finding larger and more stable benefits of demonstration than self-enactment (Allen et al., 2020; Coats et al., 2021), similar benefits of action observation and self-enactment (T.-X. Yang et al., 2024), or greater benefits of self-enactment than action observation (Lui et al., 2018). The relative benefits of each of these forms of encoding-based action effect likely reflect a combination of factors that still require systematic exploration. Each seems to offer benefits when the task requires holding instructional sequences in working memory, and the present results suggest that children obtain comparable benefits from both techniques.

Finally, consistent with previous research (Gathercole et al., 2008; Jaroslawska et al., 2016; Waterman et al., 2017; T. Yang et al., 2017, 2021) and our hypothesis, children’s ability to follow instructions was superior when recall was performed than repeated. Moreover, the interaction between encoding and retrieval was lacking, indicating that these encoding-based action advantages were similar across recall modalities in children. Specifically, children could gain benefits from motor imagery, action observation and self-enactment techniques, regardless of whether they physically enacted or verbally repeated the instructions.

## 3. Experiment 2

In this experiment, we sought to compare the performance of spoken instructions under four encoding conditions (i.e., rehearsal strategy, motor imagery, action observation, and self-enactment) in typically developing adolescents (age 12–14). To our knowledge, no prior work has investigated the ability to follow instructions in adolescent populations, and evidence regarding the benefits of action-based processing at encoding and retrieval in this population remains scarce.

Drawing on findings from adults (T.-X. Yang et al., 2024) and children (Experiment 1), we predicted superior performance in the three conditions involving action-related processing (motor imagery, action observation and self-enactment) compared with the verbal rehearsal condition. Notably, as shown in Experiment 1 involving 8- to 9-year-olds, the motor imagery benefit for instruction following is relatively weak. However, motor imagery skills improve rapidly from childhood to adolescence (Caeyenberghs et al., 2009a; Souto et al., 2020; Spruijt et al., 2015), along with the increase in working memory capacity (Gathercole et al., 2004). These patterns suggest that more cognitive resources are available for adolescents to imagine actions during encoding. Therefore, it is predicted that adolescents would show the benefits of motor imagery in following instructions. Compared with verbal rehearsal, action observation and self-enactment yield comparable mnemonic benefits in both children (Experiment 1 of the present study) and adults (T.-X. Yang et al., 2024). Accordingly, we predicted that adolescents would demonstrate similar effect sizes for the two action-based advantages.

Experiment 1 used a span procedure with varied-length instructional sequences, precluding the examination of action-based effects across serial positions. In this experiment, we therefore adopted a fixed-span instruction task with four-action sequences to enable such investigation. In young adults, prior studies have shown that the benefit of enacted recall relative to verbal recall was larger for later items in the sequence than for the earlier ones (Allen & Waterman, 2015; T. Yang et al., 2019; T.-X. Yang et al., 2024). In addition, self-enactment during encoding enhanced the verbal recall of later items in the sequence more than earlier ones (Allen & Waterman, 2015, vs. baseline without encoding instructions; T.-X. Yang et al., 2024, vs. verbal rehearsal). In contrast, evidence regarding the benefits of motor imagery and action observation across serial positions remains less clear and warrants further investigation (T.-X. Yang et al., 2024). More importantly, no study has examined encoding- and retrieval-based action advantages across serial positions in adolescents, and this gap constitutes the second goal of the present experiment.

### 3.1. Methods

#### 3.1.1. Participants

As in Experiment 1, this experiment planned to recruit 40 participants. Adolescents from a randomly selected first-grade class in a junior high school participated in the experiment. The sample included 21 girls and 19 boys, with a mean age of 12.58 years (*SD* = 0.55, from 12 to 14 years old). The present study was approved by the institute ethics committee of the corresponding author (H19052), and all the parents of participants provided informed consent.

#### 3.1.2. Design

The experiment adopted the same design as Experiment 1. In addition, as a fixed length of instructions was used in this experiment, the proportion of correct action–object pairs for each serial position was calculated (range from 0 to 1).

#### 3.1.3. The Instruction Task and Procedure

The paradigm of the instruction task was the same as Experiment 1, that is, participants listened to an action phrase, encoded it using specific methods (verbal rehearsal, motor imagery, action observation, or self-enactment), and recalled the entire sequence by oral repetition or physical enactment. We adopted the materials from a previous study involving adults (T.-X. Yang et al., 2024, Experiment 2, Lists 1–4), which used a fixed length of instructions (4 actions) and a shorter time for applying the encoding method (2.5 s). A typical instruction trial was “Shake the black bag, flip the blue folder, spin the red pencil, pull the yellow basket”.

All scoring was based on formal trials (i.e., excluding practice trials). The definition of correct action–object pairs was the same as that in Experiment 1. The way to calculate the *proportion correct of action*–*object pairs* of the list/condition was similar to Experiment 1, except the total number of correctly recalled action–object pairs was divided by 40 (i.e., the total action–object pairs in a list), leading to a score range between 0 and 1. In terms of serial position score, the number of correct action–object pairs was summed up across trials and then divided by 10 (the number of formal trials), resulting in the proportion correct of action–object pairs in that serial position (range: 0 to 1).

#### 3.1.4. Data Analysis Plan

As in Experiment 1, all analyses were carried out in JASP (https://jasp-stats.org/, accessed on 5 February 2024). The main analyses involved the 4 (encoding technique) × 2 (recall modality) repeated-measures ANOVA on the *proportion correct of action*–*object pairs*. In addition, a 4 (encoding technique) × 2 (recall modality) × 4 (serial position) mixed ANOVA was conducted on the *proportion correct of action*–*object pairs*.

### 3.2. Results

#### 3.2.1. Preliminary Analysis

There were no significant gender effects on the action–object pair scores in all conditions, with all *p* values > 0.05. The order of encoding conditions did not significantly affect action–object pair performance (*p* = 0.796). Reliability of the action–object pairs across all trials in the instruction span task was acceptable, with Cronbach’s alpha = 0.874.

#### 3.2.2. Action–Object Pairs

**Figure 3:**
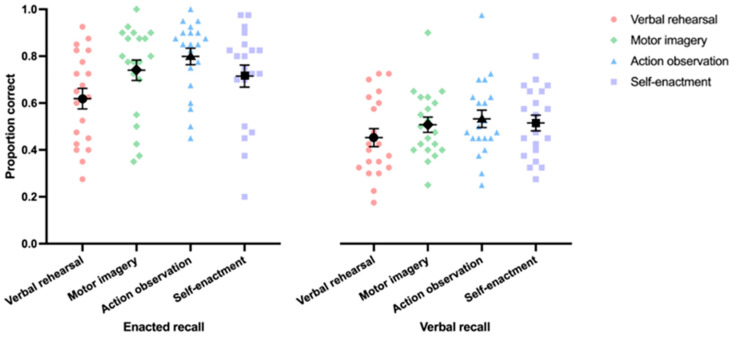
Proportion correct of action–object pairs as a function of encoding technique and recall modality in adolescents.

Descriptive results of the action–object pairs are presented in Table 1 and Figure 3. A 4 (encoding technique: verbal rehearsal, motor imagery, action observation, self-enactment) × 2 (recall modality: verbal vs. enacted) mixed ANOVA was conducted. There was a significant main effect of encoding technique, *F*(3, 114) = 7.75, *p* < 0.001, *η_p_*^2^ = 0.17, *BF*_10_ = 184.40. Post hoc analysis with Bonferroni corrections indicated a similar level of performance among the motor imagery, action observation, and self-enactment conditions, with all *p* values > 0.05, while *BF*_10_ values were from 0.18 to 0.74. Superior performance was observed in the motor imagery, the action observation and the self-enactment conditions relative to the verbal rehearsal condition, with all *p* values < 0.05, while *BF*_10_ = 10.17, 430.03, 2.32, respectively. There was a significant main effect of recall modality, showing as superior enacted recall than verbal recall, *F*(1, 38) = 24.18, *p* < 0.001, *η_p_*^2^ = 0.39, *BF*_10_ = 871.63. The interaction between recall modality and encoding technique was not significant, *p* = 0.309, *BF*_10_ = 0.96. The effect sizes of the action advantages at encoding and retrieval are presented in Table 2 and Table 3.

#### 3.2.3. Serial Position Analysis

**Figure 4:**
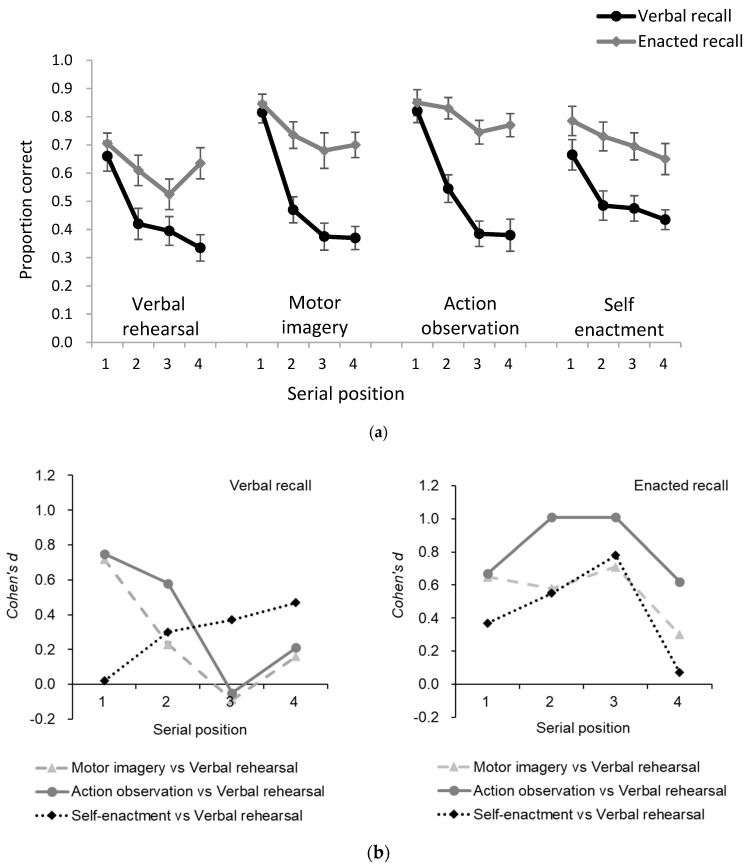
In Experiment 2 (adolescents), the upper panel (**a**) represents the proportion correct of action-object pairs as a function of serial position, encoding technique and recall modality; error bars represent standard errors. The lower panel (**b**) represents the action-based advantages during encoding across serial positions.

A 4 (encoding technique) × 2 (recall modality) × 4 (serial position) mixed ANOVA was conducted. The descriptive results are presented in Figure 4a. Here, we only report effects relating to serial position. There was a significant main effect of position, *F*(3, 114) = 60.77, *p* < 0.001, *η_p_*^2^ = 0.62, *BF*_10_ > 10,000. Position interacted significantly with encoding technique, *F*(3, 114) = 2.66, *p* = 0.005, *η_p_*^2^ = 0.07, *BF*_10_ = 6.00, and with recall modality, *F*(3, 114) = 15.18, *p* < 0.001, *η_p_*^2^ = 0.29, *BF*_10_ = > 10,000. There was also a significant three-way interaction, *F*(9, 342) = 2.25, *p* = 0.019, *η_p_*^2^ = 0.06, *BF*_10_ = 3.91.

As we focused on comparisons of encoding techniques, we further examined the interaction between encoding techniques and positions in verbal and enacted recall separately. In verbal recall, there was a significant interaction between encoding techniques and position, *F*(9, 171) = 3.11, *p* = 0.002, *η_p_*^2^ = 0.14, *BF*_10_ = 19.32. In enacted recall, there was no significant interaction between encoding techniques and position, *F*(9, 171) = 1.44, *p* = 0.173, *η_p_*^2^ = 0.07, *BF*_10_ = 0.80. To better illustrate the interaction, the effect sizes for the benefit of action-based techniques relative to verbal rehearsal (Cohen’s *d*) were calculated and are presented in Figure 4b. In verbal recall, motor imagery and action observation provided larger benefits for the first action in the sequence, whereas the benefit of self-enactment increased from the first to the last action. In enacted recall, the three action-based advantages showed a similar pattern: they offered a substantial benefit for the first three actions but a largely reduced benefit for the last action in the sequence.

### 3.3. Discussion

This experiment adopted the paradigm used in the previous study of following instructions in adults (T.-X. Yang et al., 2024), providing the first examination of this ability and the impact of different action-based encoding and retrieval mechanisms in an adolescent sample. As previously observed in adults, action-based processing at encoding or retrieval improved memory of spoken instructions in adolescents. Consistent with our hypothesis, performance on spoken instructions following was better when participants imagined performing the actions, watched videos of demonstrated actions or self-enacted during encoding, in comparison to simply rehearsing the name of the action–object pair verbally. Moreover, the three forms of action-based encoding techniques led to similar levels of performance. In other words, compared to the rehearsal strategy, the motor imagery, action observation and self-enactment strategies improved memory performance to a similar level. Enacted recall was superior to verbal recall, as consistently observed in other age groups (Allen et al., 2023). There was no strong evidence of interactions between action advantages at encoding and retrieval.

Consistent with the findings in adult studies (T.-X. Yang et al., 2024), the three action-based encoding techniques exhibited comparable memory advantages relative to verbal rehearsal. However, each has distinct strengths and limitations, and such trade-offs may result in comparable overall performance. For instance, action observation incorporates integrated visuospatial information of action–object association while lacking motor coding derived from overt action execution. Self-enactment generates motor information but also consumes limited cognitive resources. Motor imagery imposes no additional demand of physical execution yet lacks the visuospatial, motor information and action outcome information available in the former two conditions. In addition, all three action-based encoding techniques engage action planning processes in either implicit or explicit form. According to common coding theory (Prinz, 1997), action observation and action execution share overlapping representations, and neuroimaging findings further reveal that motor imagery, action observation, and action execution engage common brain network mechanisms (Hardwick et al., 2018). These findings imply that action planning may serve as a core and common cognitive process underlying the three action-based advantages, and accounting for similar memory performance in adolescents (this experiment) and adults (T.-X. Yang et al., 2024). It has been proposed that the process of action planning is rather complex, requiring individuals to link cues and movements, select motor schemas, and organize temporal frameworks (Jeannerod, 1997). Previous studies on following instructions further indicate that action planning enhances the binding of multimodal information into a coherent representation for subsequent action execution (T. Yang et al., 2016), and helps form a temporary motoric storage to maintain motoric plans briefly (Li et al., 2022).

In long-term memory research, self-enactment primarily enhances item-specific information while action observation facilitates item-relational information processing; despite this difference, both forms of action benefits stem from multimodal representation (Engelkamp, 2001). This multimodal benefit may also apply to working memory, including the following instruction task adopted in the present study. Nevertheless, important methodological differences exist between long-term and working memory paradigms. Long-term action memory tasks typically involve the free recall or recognition test of a relatively lengthy action list, whereas working memory action tasks require serial order recall of shorter action sequences. Such paradigm differences suggest that the action advantages may be underpinned by partly different mechanisms in the two memory domains. Even so, the fundamental basis of the action advantages in both domains may stem from the additional action-related information activated during encoding, which supports the formation of more integrated, multimodal, and robust memory representations for actions.

Analysis of serial position curves further revealed the mechanisms underlying instruction following and the action-based benefits. Firstly, consistent with earlier work (Allen & Waterman, 2015; T. Yang et al., 2019; T.-X. Yang et al., 2024), the serial position curves varied with recall modality. As illustrated in Figure 4a, there was a larger primacy effect in verbal than enacted recall, with a larger enacted-recall advantage at later sequence positions. This could indicate a weaker verbal-based memory representation (Allen & Waterman, 2015) or greater output interference during verbal recall (T. Yang et al., 2019). Secondly, the benefits provided by the three action-based encoding techniques varied with recall modality and serial positions; see Figure 4b. In verbal recall, imagining oneself performing the actions or observing others doing so mainly benefited memory for the first action in the sequence. This may be attributed to sufficient available cognitive resources for applying the encoding strategy, along with reduced retroactive interference for the first action in the sequence. In contrast, self-enactment tended to enhance memory for actions later in the sequence, replicating previous findings in adults (Allen & Waterman, 2015; T.-X. Yang et al., 2024), and suggesting motor memory generated by enactment is particularly useful for boosting memory of later actions when verbal representation starts to be lost. In enacted recall, the three action-based encoding techniques improved memory for action sequences in a broadly similar way. Specifically, middle actions in the sequence showed larger benefits than the last action; see Figure 4b. This might relate to the recency effect that was observed in the verbal rehearsal–enacted recall condition, that is, memory for the last action in the sequence was well-retained, thus leaving less room for further improvement by action-based encoding techniques.

Overall, these findings suggest that the action-based encoding techniques yield similar overall memory performance for spoken instructions but differ in how they facilitate memory for actions across the sequence.

## 4. General Discussion

In this study, we compared the effects of encoding techniques on following spoken instructions in both children (8 to 9 years old) and adolescents (12 to 14 years old). Overall, both children and adolescents could benefit from action-based processing at encoding and retrieval stages, and the two types of action-based benefits were additive. Specifically, participants showed higher performance when they encoded instructions via motor imagery, action observation and self-enactment than when they simply repeated the instructions verbally, although the benefit of motor imagery was weaker in children. They also had better memory performance when physically performing the actions than when orally repeating them. Since no prior study has directly compared all four encoding strategies in children or investigated instruction-following performance in adolescents, the present findings extend the enactment effects from the long-term memory literature to the working memory domain, and from adult populations to children and adolescents.

### 4.1. Action-Based Encoding Advantages Across Different Age Groups

The current work contributes to a small but growing number of studies that have directly compared action-based encoding techniques (motor imagery, action observation and self-enactment) to verbal rehearsal in improving individuals’ capacity to follow spoken instructions across age groups (children, adolescents, and adults; T. Yang et al., 2021; T.-X. Yang et al., 2024, and the present study). Overall, memory performance is improved when using action-based encoding techniques relative to using verbal rehearsal. Given the variation in instructional sequence lengths across studies, we used effect sizes (Cohen’s *d*) to quantify and compare action-based advantages across different age groups.

**Figure 5:**
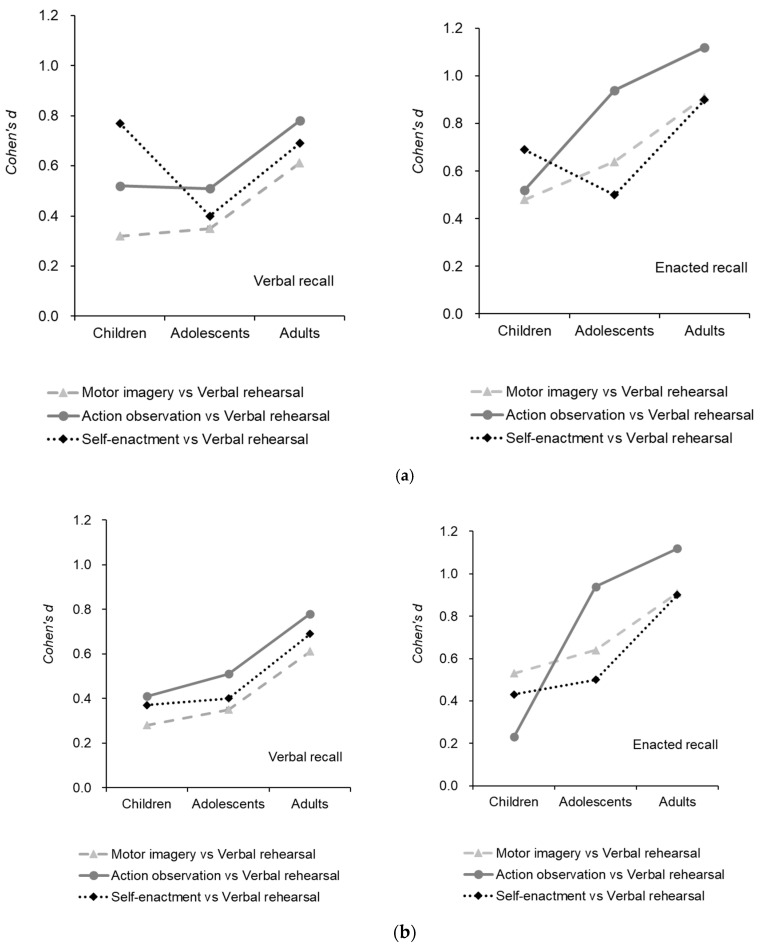
Upper panel (**a**) represents the effect sizes of action advantages (Cohen’s *d*) in children and adolescents (Experiments 1 and 2 of present study), and in adults (T.-X. Yang et al., 2024). Note the paradigms of following instructions were different for children (involving 2- to 4-action sequences) and for adolescents and young adults (including 4-action sequences). Lower panel (**b**) represents the effect sizes of action advantage (Cohen’s *d*) in children (Experiment 1 of the study, only 4-action sequences were selected), adolescents and adults (4-action sequences).

As illustrated in Figure 5a, the benefit of motor imagery relative to verbal rehearsal increases from childhood to adulthood, which may relate to the development of motor imagery skills (Fernández García et al., 2021; Spruijt et al., 2015) and working memory (Alloway & Alloway, 2013), as both abilities contribute to forming and maintaining a more refined and robust action representation. The benefit of action observation relative to verbal rehearsal also increases with age. Since visuomotor processes are critical for this advantage (Allen et al., 2020) and the ability to process and maintain visuospatial–motor information in working memory improves with age (Gathercole et al., 2004), individuals are more likely to learn from viewing others’ demonstrations as they grow older.

In contrast, the self-enactment benefit was smaller in adolescents than in children; see Figure 5a. The reason for this is unclear, and it may simply reflect methodological differences between the two experiments. One speculation is that the longer instructions and shorter duration for self-enactment during encoding for adolescents (4-action sequences, 2.5 s for self-enactment) than for children (2-, 3- or 4-action sequences, and 3 s for self-enactment) might have increased task difficulty and reduced the potential benefit of self-enactment in adolescents. To validate this possibility, the action-based benefit of four-action sequences in children (Experiment 1^1^) was calculated to compare with that in adolescents (Experiment 2) and adults (T.-X. Yang et al., 2024). As shown in Figure 5b, the self-enactment benefit in children was largely reduced, verifying that the benefit may be contingent on instruction length, which requires further validation.

These findings are in line with previous evidence that in 6–10-year-old children, the self-enactment benefit relative to no-enactment only emerged under simpler instructions. In fact, performance in the self-enactment condition was worse than in the no-enactment condition when instructions were complex (Waterman et al., 2017). These findings suggest that self-enactment is sensitive to the cognitive load of the memory task. Indeed, when tasks are complex, self-enactment can be distracting and consume limited cognitive resources, particularly when action sequences are novel and participants have less experience with action planning. In relation to long-term memory, Steffens et al. (2015) also indicated that self-enactment during encoding may divert participants’ attention away from memorizing the retrieval routes. Together, these findings suggest the self-enactment effect is conscious and goal-directed rather than implicit or non-strategic (Roberts et al., 2022, a review).

It is worth noting that the action observation advantage in children was also reduced when instructional sequences were longer, especially for enacted recall, implying that the benefit afforded by demonstration also requires attention (see Figure 5). As Steffens et al. (2015) noted, people often assume that learning by doing (i.e., self-enactment) is superior to learning by viewing (i.e., demonstration), because they tend to spend less attention and mental effort when watching others’ demonstrations than performing the task by themselves.

However, caution should be taken as these inferences were based on comparisons of effect sizes from varying instruction-following paradigms across studies. Future research may consider systematically investigating the effect of cognitive load on these action-based benefits across working memory and long-term memory domains, and compare such benefits in following spoken instructions across the lifespan using consistent paradigms.

### 4.2. Mechanisms Underlying Encoding-Based Action Advantages in Working Memory

The three forms of action-encoding advantage currently under investigation (i.e., motor imagery, action observation, and self-enactment) share similarities and differences. They all incorporate action-related information and transform purely verbal representations of spoken instructions into multimodal memory representations, which explains their comparable facilitative effects in certain experiments (Wojcik et al., 2011, children with autism; T.-X. Yang et al., 2024; Experiment 2 of the present study). In each case, they likely draw on a collection of different forms of representation and control. These dimensions can be broadly mapped onto the multicomponent working memory framework (Allen et al., 2026; Hitch et al., 2025; Baddeley et al., 2021), with verbal instruction loading on the phonological loop, visuospatial processing involving the visuospatial sketchpad, and strategic effortful control drawing on central executive resources. We assume that motor coding is also involved to a varying extent in each action condition. However, this is not an explicit and distinct subsystem within the multicomponent model. Based on motor dual-task interference, Jaroslawska et al. (2018) suggested the addition of a separate motor store in working memory that could temporarily hold the results of motoric processing. Li et al. (2022) partly replicated and extended the findings of Jaroslawska et al. (2018), but they argued that motor coding could be incorporated within a broader conception of the visuospatial sketchpad rather than as a distinct and specialized store.

Turning to other theoretical approaches, the Time-Based Resource-Sharing (TBRS) approach of Barrouillet and Camos (2020) proposes several peripheral subsystems that temporarily hold modality-specific information, including phonological, visuospatial, and motor buffers. Cowan’s embedded processes framework (Cowan et al., 2020) would also allow for motor coding in working memory tasks, though this perspective views such representations as temporarily activated long-term memory. Recently, McDougle and Hillman (2025) have also set out the concept of a motor working memory store, reflecting a distributed neural circuit including the sensorimotor cortex and the prefrontal cortex. They suggest that various forms of action coding, including imagery, could be captured within this motor working memory subsystem, though they do not attempt to place this within a broader working memory framework.

Regarding the multimodal aspect of these action advantages, recent developments in an updated iteration of the multicomponent model of working memory are highly relevant (Allen et al., 2026). This model considers storage buffers as representing confluences of input from different sources, with the combination of different forms of information being consciously accessible to the participant and available to support recall via the awareness buffer. On a similar note, Cowan et al. (2025) propose the funneling of information from various cognitive domains into a conscious whole, captured within the focus of attention that is centered within the parietal lobe. Thus, within each of these working memory perspectives, verbal, visuospatial, and motoric information are registered as distinct and specialized forms of storage, and cohere as consciously accessible multimodal representations in the awareness buffer/focus of attention.

Of course, the three forms of action-encoding advantage also likely incorporate unique features. For instance, motor imagery requires individuals to internally generate action plans without physical execution; action observation acquires direct visual input of integrated action representation linking objects and actions and thus eliminates the need for endogenous action planning; and self-enactment involves both internal plan generation and actual physical manipulation, producing extra motor codes plus outcome feedback. These processing discrepancies may place different demands on subcomponents of working memory and consequently lead to varied action advantages across studies (Allen et al., 2020; Coats et al., 2021; Lui et al., 2018; Waterman et al., 2017; Wojcik et al., 2011, typical children). For instance, the serial position curves of the three forms of action-based encoding conditions were different in adolescents in the present study, in contrast to similar patterns in adults (T.-X. Yang et al., 2024), suggesting working memory capacity may influence the extent to which these advantages can be attained.

As noted by McDougle and Hillman (2025), questions of action and motor processing in working memory have been neglected and more work is needed. Future research regarding action-based effects in working memory could advance along various directions. For example, neuroimaging approaches could help unpack neural mechanisms differentiating the three forms of action-based encoding. From a cognitive-processing perspective, additional indices including reaction time and feature binding might be adopted to explore temporal dynamics and object–action integration underlying the different encoding-based action advantages that have been observed in working memory in the current study. In the future, it could be informative to develop a paradigm to examine action-based effects in both working memory and long-term memory simultaneously. This could support the development of a unitary model or framework to capture enactment effects across working memory and long-term memory. Such advances would deepen our understanding of action-based advantages and facilitate the development of an integrated theory to explain enactment benefits in both memory systems.

### 4.3. Contributions and Limitations

Previous studies have only compared the effects of two or three types of encoding strategies for children’s performance on instruction-following tasks (Wojcik et al., 2011; Xie et al., 2022; T. Yang et al., 2021), with no research having simultaneously compared all four encoding methods (i.e., verbal rehearsal, motor imagery, action observation, self-enactment). Moreover, there had been no study specifically examining instruction following and action advantages in adolescents. The present study is the first to directly compare all four typical encoding techniques (verbal rehearsal, motor imagery, action observation and self-enactment) in both children and adolescents. Moreover, the analysis of serial position curves revealed that different mechanisms might underlie different action-based advantages at encoding in adolescents. Furthermore, this study integrates the latest working memory theories to provide a preliminary framework for explaining the mechanisms underlying these encoding-based action advantages, and points to directions for future research.

From an applied perspective, the current results indicate that teachers in primary and junior schools might consider implementing action-based techniques to improve students’ instruction-following ability where appropriate and suitable for the context. Given their similar effectiveness but unique advantages and application contexts, teachers can select appropriate methods accordingly. For instance, motor imagery and action observation are more suitable than self-enactment when an action sequence cannot be executed immediately after each instruction step. In addition, the present findings suggest that motor imagery may not consistently improve memory for instructions in 7- to 8-year-old children; instead, self-enactment and action demonstration techniques might be better options.

The present study has several limitations. Firstly, we used different instructional materials for children and adolescents, which precluded statistical comparisons between the two groups. Future research should use a wider range of instruction spans (e.g., from span 2 to 6) to examine age-related changes in action-based advantages. Secondly, we did not include a baseline condition in which participants could freely choose encoding strategies, and future work should compare the four instructed encoding conditions with this baseline condition to reveal their effectiveness relative to no requirement on strategies.

### 4.4. Conclusions

Overall, encoding techniques involving motor processing (motor imagery, action observation, and self-enactment) outperform verbal rehearsal in aiding children and adolescents to follow spoken instructions, although motor imagery yields a slightly weaker benefit in children. These findings provide empirical support for implementing these techniques in daily teaching and learning practice, though further work is clearly needed to explore how these methods might be best incorporated.

## Figures and Tables

**Table 1:** Descriptive results of action–object pairs in Experiment 1 (children) and Experiment 2 (adolescents).

	Verbal Recall *M* (*SD*)	Enacted Recall *M* (*SD*)
Children	(*N* = 20)	(*N* = 19)
Verbal rehearsal	0.46 (0.15)	0.57 (0.19)
Motor imagery	0.50 (0.14)	0.65 (0.16)
Action observation	0.53 (0.12)	0.65 (0.14)
Self-enactment	0.57 (0.14)	0.68 (0.13)
Adolescents	(*N* = 20)	(*N* = 20)
Verbal rehearsal	0.45 (0.17)	0.62 (0.20)
Motor imagery	0.51 (0.15)	0.74 (0.20)
Action observation	0.53 (0.16)	0.80 (0.17)
Self-enactment	0.52 (0.15)	0.72 (0.21)

**Table 2:** Action advantages at encoding stage (Cohen’s *d*) in Experiment 1 (children) and Experiment 2 (adolescents).

	Verbal Recall	Enacted Recall
Children	(*N* = 20)	(*N* = 19)
Motor imagery vs. verbal rehearsal	0.32	0.48
Action observation vs. verbal rehearsal	0.52	0.52
Self-enactment vs. verbal rehearsal	0.77	0.69
Action observation vs. motor imagery	0.20	0.05
Self-enactment vs. motor imagery	0.45	0.21
Self-enactment vs. action observation	0.25	0.16
Adolescents	(*N* = 20)	(*N* = 20)
Motor imagery vs. verbal rehearsal	0.35	0.64
Action observation vs. verbal rehearsal	0.51	0.94
Self-enactment vs. verbal rehearsal	0.40	0.50
Action observation vs. motor imagery	0.16	0.31
Self-enactment vs. motor imagery	0.05	−0.13
Self-enactment vs. action observation	−0.11	−0.44

*Note.* Positive effect sizes represent higher performance in the former than the latter condition, and the negative effect sizes indicate the opposite.

**Table 3:** Action advantages at retrieval stage (Cohen’s *d*) in Experiment 1 (children) and Experiment 2 (adolescents).

Children	(*N* = 39)
Enacted-recall advantage in verbal rehearsal condition	0.80
Enacted-recall advantage in motor imagery condition	0.95
Enacted-recall advantage in action observation condition	0.80
Enacted-recall advantage in self-enactment condition	0.71
Adolescents	(*N* = 40)
Enacted-recall advantage in verbal rehearsal condition	0.90
Enacted-recall advantage in motor imagery condition	1.35
Enacted-recall advantage in action observation condition	1.64
Enacted-recall advantage in self-enactment condition	1.10

## Data Availability

Data can be acquired through this link: 10.57760/sciencedb.psych.00795, accessed on 12 May 2026.

## Note

1 Performance of four action sequences as a function of encoding technique and recall modality in children are presented in the Supplementary Materials.

## Supplementary Materials

The following supporting information can be downloaded at: https://www.mdpi.com/article/10.3390/bs16061008/s1. Table S1. Descriptive results of 4-action sequences in children (Experiment 1). Table S2. Descriptive results of prediction, performance and postdiction in children (Experiment 1) and adolescents (Experiment 2). Figure S1. Proportion correct of action-object pairs for the four-action sequences as a function of serial position, encoding technique and recall modality in children (Experiment 1). Reference (Godfrey et al., 2023) is cited in the Supplementary Materials.
