# Supplementary material for: Action-Based Encoding Improves Instruction Following in Children and Adolescents
Source: Behav Sci (Basel). 2026 Jun 16;16(6):1008. doi: 10.3390/bs16061008 (PMC13295551; doi:10.3390/bs16061008)
Supplement: Supplementary file 1 [file behavsci-16-01008-s001.zip › behavsci-4266696-supplementary.pdf]

Supplementary materials

1. Performance of 4-action sequences in children (Experiment 1)

To ensure consistency in instruction length between children (Experiment 1) and adolescents (Experiment 2), four-action sequences from the Span task in Experiment 1 were selected. Each list/condition comprised of four sequences.

The descriptive results of proportion correct of action-object pairs in children are presented in Table S1. The 4 (encoding technique: verbal rehearsal, motor imagery, action observation, self-enactment) × 2 (recall modality: verbal vs. enacted) ANOVA indicated a significant main effect of encoding technique, F (3, 111) = 2.77, p = 0.045, $\eta_p{}^2$ = 0.07, BF_{10} = 0.61. Post-hoc analyses with Bonferroni corrections suggested a trend of higher performance of the motor imagery condition compared with the verbal rehearsal condition, p = 0.075, Cohen's d = 0.44, BF_{10} = 2.96, whereas the performance of the action observation and self-enactment conditions were similar to the verbal rehearsal condition, p = 0.388, p =.100, respectively. There was no significant difference among the three action-based encoding strategies, all p values > .05. The main effect of recall modality was significant, F (1, 37) = 8.20, p = 0.007, $\eta_p{}^2$ = 0.18, BF_{10} = 4.97. The interaction between encoding technique and recall modality was not significant, p = 0.537.

Table S1. Descriptive results of 4-action sequences in children (Experiment 1)

	Verbal recall<br>*M (SD)*	Enacted recall<br>*M (SD)*
**Action-object pairs**		
Verbal rehearsal	0.24 (0.18)	0.36 (0.29)
Motor imagery	0.29 (0.18)	0.50 (0.24)
Action observation	0.31 (0.17)	0.42 (0.24)
Self-enactment	0.31 (0.20)	0.47 (0.23)

The serial position curves of four-action sequences in children are presented in Figure S1. The 4 (encoding technique) × 2 (recall modality) × 4 (serial position) mixed ANOVA was conducted and we only focused on results relating to serial positions. There was a significant main effect of serial position, F (3, 111) = 9.37, $p <$ 0.001, $\eta_p{}^2 = 0.20$. Serial position did not interact with recall modality or encoding technique, and there was no three-way interaction, all p values > 0.05.

Figure S1

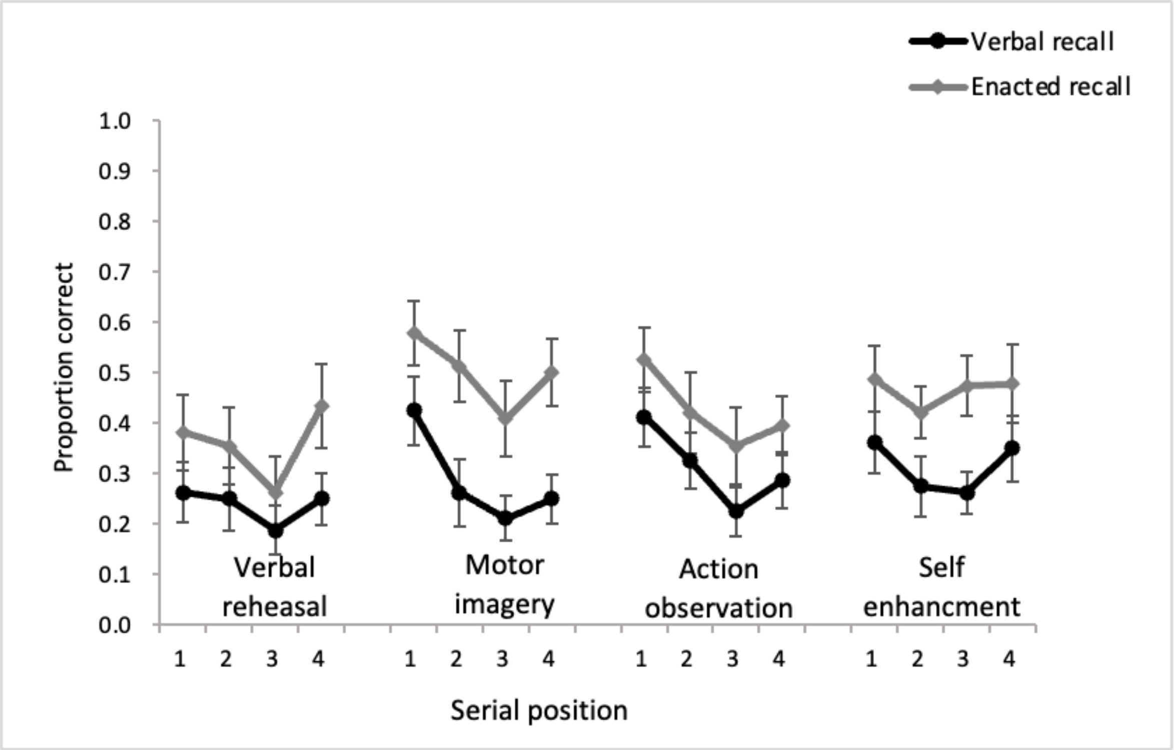

Fig S1. Proportion correct of action-object pairs for the four-action sequences as a function of serial position, encoding technique and recall modality in children (Experiment 1). *Note.* Error bars represent standard errors.

To compare children and adolescents' performance on four-action sequences, the data of Experiment 1 and 2 were combined. The 4 (encoding technique: verbal rehearsal, motor imagery, action observation, self-enactment) × 2 (recall modality: verbal vs. enacted) × 2 (age group: children vs. adolescents) mixed ANOVAs analysis on proportion correct of action-object pairs was conducted. Here, we only reported age-related results. The main effect of age group was significant, with

adolescents outperforming children, F (1, 75) = 52.59, p < 0.001, η_p^2 = 0.41, BF_{10} > 10000. However, all the age-related two-way or three-way interactions were not significant, all p values > 0.05.

In summary, 8- to 9-year-old children exhibited lower performance than 12- to 14-year-old adolescents when following 4-action sequences. For children, the encoding-based action advantages in the span procedure (including 2 to 4 actions) were less evident in 4-action sequences. These findings suggest that when spoken instructions were longer and more challenging for children to encode and retain, the encoding-based action advantages diminished. Practically, the results imply that action-encoding techniques are optimally applied in scenarios where instruction length is within children's working memory capacity.

2. Prediction, evaluation and performance of following instructions in children and adolescents

In both experiments, participants were required to make predictions and postdictions of their performance. In each condition, participants were required to complete two practice trials involving 2-action sequences. Next, children in Experiment 1 were told that they would begin with 2-action sequences and move on to longer sequences, and the adolescents in Experiment 2 were told that they would be tested using the 4-action sequences. Then, all the participants were required to predict their performance on a 0 to 100 scale, with higher scores representing better performance. Immediately after completion of the condition, participants were asked to rate their actual performance using the 0 to 100 scale.

Given different instruction lengths were used for children and adolescents, separate analyses were conducted for each sample. First, the proportion correct of action-object pairs (range: 0-1) was transformed to performance score (range: 0-100). Second, the ratings of prediction, postdiction and performance were compared, and the effects of encoding technique and recall modality were examined. The 4 (encoding technique: verbal rehearsal, motor imagery, action observation, self-enactment) $\times$ 2 (recall modality: verbal vs. enacted) $\times$ 3 (score type: prediction,

performance, postdiction) mixed ANOVA analyses were conducted, focusing on the effect of score type and its interaction with the other two factors. Finally, the effects of encoding technique and recall modality in the prediction score and the postdiction score were examined, using the 4 (encoding technique: verbal rehearsal, motor imagery, action observation, self-enactment) $\times$ 2 (recall modality: verbal vs. enacted) mixed ANOVA analyses.

Results in children

The descriptive results of children are presented in Table S2. The main effect of score type was significant, F (2, 74) = 21.74, p < 0.001, η_p^2 = 0.37, showing as higher prediction score and postdiction scores compared with the performance score, both *p values* < 0.001; whereas the prediction score and the postdiction score were similar, p = 0.232. The score type did not interact with encoding technique or recall modality, p = 0.832.

Prediction score. The main effect of encoding technique was not significant, p = 0.308, The main effect of recall modality was significant, F (1, 37) = 4.71, p = 0.036, η_p^2 = 0.11, with children predicting higher performance for enacted than verbal recall. The interaction between encoding technique and recall modality was not significant, p = 0.832.

Postdiction score. The main effect of encoding technique was not significant, p = 0.186, The main effect of recall modality was marginally non-significant, F (1, 37) = 3.69, p = 0.062, η_p^2 = 0.09, with children showing a trend for reporting better performance for enacted recall than for verbal recall. The interaction between encoding technique and recall modality was not significant, p = 0.925.

Table S2. Descriptive results of prediction, performance and postdiction in children (Experiment 1) and adolescents (Experiment 2)

	Experiment 1 (children)		Experiment 2 (adolescents)	
	Verbal recall *N= 20* *M (SD)*	*Enacted recall* *N=19* *M (SD)*	*Verbal recall* *N= 20* *M (SD)*	*Enacted recall* *N=20* *M (SD)*
**Prediction**				
Verbal rehearsal	66.85 (29.53)	82.74 (19.39)	70.90 (18.93)	66.10 (20.03)
Motor imagery	65.30 (28.73)	82.74 (13.31)	69.85 (19.02)	67.55 (17.54)
Action observation	70.45 (31.12)	83.11 (18.63)	72.25 (18.14)	71.65 (18.64)
Self-enactment	72.75 (26.87)	85.79 (17.67)	65.20 (16.97)	69.60 (20.08)
**Performance**				
Verbal rehearsal	45.60 (14.47)	57.42 (19.45)	45.25 (17.09)	61.88 (19.62)
Motor imagery	50.40 (14.12)	64.47 (16.39)	50.75 (14.49)	74.00 (19.52)
Action observation	53.30 (12.22)	65.16 (13.91)	53.25 (16.65)	79.88 (15.76)
Self-enactment	57.00 (13.66)	67.58 (13.44)	51.50 (14.99)	71.50 (21.02)
**Postdiction**				
Verbal rehearsal	62.80 (29.26)	77.68 (19.86)	61.40 (22.07)	59.95 (22.85)
Motor imagery	60.75 (28.19)	75.40 (27.05)	58.30 (24.51)	62.55 (20.17)
Action observation	64.60 (27.95)	79.26 (18.17)	65.20 (16.97)	69.60 (20.08)
Self-enactment	67.90 (24.33)	79.63 (21.48)	62.85 (20.71)	59.10 (23.27)

Results in adolescents

The descriptive results are presented in Table S2. Here, we only report the main effect of score type, and its interactions with other factors. The main effect of score type was significant, F (2, 76) = 6.56, p = 0.002, η_p^2 = 0.15, with significantly higher prediction score compared with the performance score, p = 0.004, and with the postdiction score, p = 0.018, whereas there was no significant difference between the performance score and the postdiction score, p = 1.000. The score type significantly interacted with encoding technique, F (6, 228) = 2.22, p = 0.042, η_p^2 =

0.06, reflecting as larger differences between the prediction and performance score in the verbal rehearsal condition than in the other encoding conditions. The score type also significantly interacted with recall modality, F (2, 76) = 13.69, $p < 0.001$, $\eta_p^2 = 0.27$. The enacted-recall advantage was significant in the performance score, $p = 0.001$, but not in the prediction score and the postdiction score, both p values = 1.000. In contrast, overestimation of performance during prediction and postdiction was significant only for verbal recall conditions, $p < .001$, $p = 0.022$, respectively, but not for enacted recall, both p values = 1.000.

Prediction score. The main effect of encoding technique, recall modality and their interaction were not significant, $p = 0.418$, $p = 0.579$, $p = 0.828$, respectively.

Postdiction score. The main effect of encoding technique was significant, F (3, 114) = 3.23, $p = 0.025$, $\eta_p^2 = 0.08$, as adolescents tended to evaluate better performance in the action observation condition than in the verbal rehearsal condition $p = 0.074$, and the motor imagery condition, $p = 0.057$. The main effect of recall modality, and its interaction with encoding technique were not significant, $p = 0.886$, $p = 0.311$, respectively.

Discussion

In both pre-task predictions and post-task evaluations, children overestimated their performance, reflecting overconfidence in their ability to follow spoken instructions and a lack of post-performance adjustment; this pattern was unaffected by encoding technique or recall modality. In addition, their prediction and postdiction scores were modulated by recall modality but not encoding conditions, suggesting that children were more sensitive to action-based advantages at the retrieval stage than at encoding. A limitation of the study was that children were required to make the prediction after completing the 2-action sequence practice. Despite being informed of increasing instruction length in the test phase, children may have anchored their predictions to the simpler 2-action practice performance, failing to consider increased difficulty as instruction length increased. Moreover, children displayed persistent overestimation of actual performance post-task, suggesting the

bias of prediction may not be fully attributed to unrepresentative practice before prediction.

Similar to children, adolescents overestimated their performance during prediction, with this overconfidence potentially attributed to anchoring predictions in 2-action practice performance-failing to account for increased difficulty in following longer sequences despite advance notification of 4-action instructions. In addition, adolescents' predictions remained unmodulated by encoding condition, suggesting insensitivity to encoding-based action advantages. Unlike children, adolescents demonstrated post-performance evaluative adjustment: their postdiction scores more accurately mirrored actual performance. Adolescents also reported superior postdiction on performance of the action-observation condition relative to verbal rehearsal and motor imagery conditions, suggesting an awareness of potential action-observation benefits post-application, despite equivalent actual performance across these conditions. Concerning the enacted-recall advantage, while children predicted this benefit, adolescents did not, which reflects adolescents' overconfidence in verbal recall performance.

Overall, these findings support previous studies reporting children's overconfidence in their memory abilities, and the development of metamemory from childhood to adolescence (Godfrey et al., 2023). Additionally, this study provides novel evidence of overconfidence in children and adolescents during prediction and evaluation of a novel action-based working memory task. Notably, this study reveals that adolescents, unlike children, demonstrated the capacity to adjust their post-task judgments about performance to align with how they had actually performed on the task.

References

Godfrey, M., Casnar, C., Stolz, E., Ailion, A., Moore, T., & Gioia, G. (2023). A review of procedural and declarative metamemory development across childhood. *Child Neuropsychology*, *29*(2), 183-212. https://doi.org/10.1080/09297049.2022.2055751
